# The Relationship Between Asthma and Food Allergies in Children

**DOI:** 10.3390/children11111295

**Published:** 2024-10-26

**Authors:** Daniela Cunico, Giuliana Giannì, Sara Scavone, Enrico Vito Buono, Carlo Caffarelli

**Affiliations:** Clinica Pediatrica, Department of Medicine and Surgery, University of Parma, 43125 Parma, Italy; daniela.cunico@unipr.it (D.C.); giuliana.gianni@unipr.it (G.G.); sara.scavone@unipr.it (S.S.); enricovito.buono@unipr.it (E.V.B.)

**Keywords:** asthma, food allergy, allergic march, anaphylaxis, omalizumab, oral immunotherapy, children, biologics

## Abstract

Asthma and food allergy are two complex allergic diseases with an increasing prevalence in childhood. They share risk factors, including atopic family history, atopic dermatitis, allergen sensitization, and T2 inflammatory pathways. Several studies have shown that in children with a food allergy, the risk of developing asthma, particularly in early childhood, is high. Food allergen intake or the inhalation of aerosolized allergens can induce respiratory symptoms such as bronchospasm. Patients with both conditions have an increased risk of severe asthma exacerbations, hospitalization, and mortality. The current management of clinical food hypersensitivity primarily involves the dietary avoidance of food allergens and the use of self-injectable adrenaline for severe reactions. Poorly controlled asthma limits the prescription of oral immunotherapy to foods, which has emerged as an alternative therapy for managing food allergies. Biological therapies that are effective in severe asthma have been explored for treating food allergies. Omalizumab improves asthma control and, either alone or in combination with oral immunotherapy, increases the threshold of allergen tolerance. Understanding the interplay between asthma and food allergy is crucial for developing successful treatment approaches and ameliorating patient results.

## 1. Introduction

The link between allergic diseases is well known, in particular between asthma and food allergies. In recent decades, the prevalence of asthma has been assessed in several studies [1]. The global prevalence of asthma in childhood was approximately 9% of children in 1998 [2], with a great variation worldwide. The frequency ranged from 1% to 30% between countries and was higher in Western and English-speaking countries. The prevalence among 13–14-year-olds ranged from 2.1% in Indonesia to 32.2% in the United Kingdom. The prevalence of wheezing reported by parents in 6- to 7-year-olds ranged from 4.1% in Indonesia to 32.1% in Costa Rica. The prevalence of asthma increased to 11.5% in the phase III ISAAC study (2001–03), with differences among centers of the same nation [3]. Using the same questionnaire as ISAAC, in 2020, the Global Asthma Network (GAN) phase I study showed that the global asthma prevalence was 10.4%, and it did not change in Western countries but increased in developing countries [4]. Regarding food allergy, in a meta-analysis of studies published between 1 January 2000 and 30 June 2021 conducted by Spolidoro et al., the self-reported prevalence of food allergy in Europe among 2–5-year-olds ranged from 1.6 to 38.7% and from 1.6 to 47.5% in children between 6 and 17 years old. However, food allergy proven by oral challenge provided lower prevalence estimates, ranging from 0 to 4.2% in children between 2 and 5 years old, and from 0 to 5.7% among 6–17-year-olds. The lifetime and point prevalence of self-reported food allergy overall stood at 20% and 13%, respectively. Based on clinical history or a positive food challenge result, the prevalence of food allergy increased from 2.6% in 2000–2012 to 3.5% in 2012–2021 [5].

Asthma and food allergies are two complex T2-mediated diseases, in which both environmental and genetic factors play a role. They share similar risk factors (i.e., parental or family history of allergy, atopic dermatitis, and allergen sensitization), and several studies have demonstrated that children with food allergies have a higher risk of asthma occurrence. An association between asthma and non-IgE-mediated food allergies has also been observed [6]. This review aims to bridge the gaps in the current understanding of the interactions between food allergies and asthma from a clinical point of view. Moreover, we intend to fill the knowledge gaps regarding new treatments used both for food allergies and asthma. We therefore conducted a literature search using the PubMed database to gather information, using keywords such as “food allergy”, “asthma”, “biologics”, and “children” to retrieve articles. Articles published in the last 10 years up to April 2024 and written in English were searched. We considered only significant articles for the purpose of this review. In addition, relevant articles that authors knew or recognized in the references of the selected articles were considered.

## 2. Asthma–Food Allergy Comorbidity

### 2.1. Co-Morbidity Prevalence and Temporal Development

Around 48% of subjects with asthma have food allergies, and about half of children with food allergies have allergic respiratory symptoms. In almost 45% of asthmatic people, a sensitization for at least one food item such as egg, cow’s milk, soy, peanut, wheat, and fish is reported, with a higher prevalence than in non-asthmatic patients [1]. Children with IgE-mediated food allergies are at risk for respiratory allergy [7]. A positive skin prick test response to egg at 3 months, 6 months, or 1 year was a predictor of asthma in a 22-year follow-up study [8]. Food sensitization in the first 2 years of life was associated with an increased risk of asthma and allergic rhinitis by 10–12 years of age, regardless of sensitization to inhalants [9]. Hypersensitivity to food allergens is linked with persistent asthma (30.4%) at age 24 years [10]. Consequently, the modified asthma predictive index (mAPI), a score used to define patients most likely to develop persistent asthma, includes IgE to milk, egg, and peanuts among the minor criteria [11]. This relation seems even stronger in patients who are sensitized to foods in the early years of life or have persistent allergies [12,13]. On the other hand, asthma prevalence in children with food allergies is increased (up to two–threefold) compared to people without food allergies [7,9,14,15,16,17]. Children allergic to cow’s milk show a higher prevalence of asthma at 15 and 26 years of age [18]. This indicates that food allergies predispose asthma onset not only in childhood but also in adulthood. Traditionally, the transition from food allergy to asthma is considered in the context of the so-called atopic march. The atopic march defines the natural temporal sequence of allergic diseases mediated by Th2 lymphocytes over infancy and childhood. Initially, the allergic march did not include IgE-mediated food allergy, but it soon became clear that it was an important element. The classical “atopic march” was based on population studies that have shown that the peak occurrence of food sensitization, food allergies, and atopic dermatitis was in the first 2 years of life, and it preceded that of aeroallergen-specific IgE responses, asthma, allergic rhinitis, and allergic conjunctivitis [12,19]. In fact, very frequently, children suffering from an allergic disease develop other allergic diseases. However, more recent insights have shown that the natural history of allergies in children can follow heterogeneous trajectories, rather than single-sequential development starting with eczema and food allergies, progressing with asthma, and ending with rhinitis, as previously thought [20,21]. The progression from atopic dermatitis to respiratory allergy (asthma or allergic rhinitis) has been found to be the predominant trajectory, occurring in about 12% of cases [22], followed by atopic dermatitis with the subsequent development of food allergies. An IgE-mediated food allergy, especially to cow’s milk, peanut, and hen’s egg, is frequently the alternative entry point and it is followed by asthma or allergic rhinitis [23]. It is of note that about 15–50% of children have isolated atopic dermatitis and 5.7% have isolated wheeze without atopic dermatitis, indicating the absence of the atopic march [22,23].

### 2.2. Risk Factors

The basis for the coexistence and heterogeneous trajectories of atopic diseases is unclear. It reflects not only the crucial immunologic mechanisms that induce a Th2 response following allergen exposure [24] but also genetic and environmental factors. Multiple genes contribute to asthma development [25] and subtypes of asthma have distinct genetic associations [26,27]. It has been shown that asthma, hay fever, and eczema share genetic loci [28]. A genome-wide association study [23] found that atopic dermatitis in Asian and Pacific Islanders was associated with IgE food allergy development, while single-nucleotide polymorphisms were associated with the development of asthma or rhinitis in patients with atopic dermatitis of European ancestry or African ancestry. Variants of several genes are associated with the risk of both IgE-mediated food allergy and asthma, including C11orf30 [29,30], STAT6 [31], and thymic stromal lymphopoietin (TSLP) [23]. The risk of the onset of both asthma [32] and food allergy [33] is increased by exposure to industrial- and traffic-related air pollution and indoor air pollution, including smoke exposure in utero and after birth, to which Black children are more exposed. Of note, environmental factors can act by epigenetic modifications, especially through DNA methylation [34], and microbiome alterations. According to the microbial diversity hypothesis, altered gut microbiota can predispose an individual to both asthma and food allergy by modifying the immune response through trained immunity mechanisms that include epigenetic modifications. Along this line, an increased risk of asthma has been shown to be associated with a higher abundance of several microbial genera [35,36]. Environmental factors that modify the gut microbiome, including vaginal delivery, maternal milk, and the intake of antibiotics or antacids, increased the risk of atopic dermatitis, IgE-mediated food allergy, asthma, and allergic rhinitis during childhood [37]. Moreover, gut microbiome dysregulation can reduce food tolerance [38] and predispose an individual to asthma [23,39]. However, further studies are warranted to determine whether genetic and environmental factors can influence allergic trajectories. These studies should consider the potential additional effect of lifestyle and socioeconomic status. A sedentary lifestyle and unhealthy Western diet may lead to obesity, which favors asthma onset [40]. Obese asthmatics have reduced asthma control, low quality of life, and more frequent emergency room visits and hospitalizations [41]. The Western diet is characterized by an elevated intake of saturated fats. Fatty acids worsen airway inflammation in asthmatics [42]. However, in a randomized controlled study, Lang [43] found that N-3 polyunsaturated fatty acids supplementation did not improve asthma control and lung function at 3 and 6 months in adolescents and young adults. Similarly, the results of studies on polyunsaturated fatty acid supplementation during pregnancy and lactation on the prevention of food allergy are contradictory [44], even if in animal models [45]. N-3 long-chain polyunsaturated fatty acids protect from the development of food allergies. Regarding socioeconomic status, low-income patients are more exposed to outdoor and indoor environmental factors that favor the onset of asthma because of a lack of green spaces and living in houses with molds, allergens, and moisture. They have limited access to care and to specialty care, with underdiagnosis and undertreatment of allergic diseases, including asthma and food allergies [46]. They also have increased asthma morbidity and poor asthma control with an increased use of healthcare services [47,48]. Furthermore, children of low socioeconomic status with food allergies utilising food assistance programs have an increased risk of accidental intake of the offending food [49].

### 2.3. Severity of Symptoms in Subjects with Asthma and Food Allergy

The coexistence of asthma and food allergy in the same individual may increase the severity of both conditions. IgE directed against cross-reactive allergens may be responsible for both respiratory reactions and allergic reactions to food.

#### 2.3.1. Food Allergy and Lung Function

Food allergies and sensitization to a particular food at an early age appear to be associated with decreased lung function. Sherenian MG et al. [50] analyzed a population of 1068 patients, of which 403 had food allergies and 407 had asthma. In patients with asthma, a significant reduction in forced expiratory flow (FEF) of 25–75% was highlighted only in patients with two or more food allergies, while no differences were found for forced expiratory volume (FEV) 1, forced vital capacity (FVC), or FEV1/FVC between patients with or without food allergies [50]. Peters et al. [51] also analyzed a large population of 5276 children. They found that children with food allergies at one year of life had a reduction in FEV1 (−0.19 [95% CI: 0.32 to −0.06] and FVC (−0.17 95% CI: [−0.31 to −0.04]) at the 6th year of life, and also that both patients with sensitization and patients with food allergy had a greater risk of asthma development at 6 years [51]. Moreover, Friedlander JL et al. found a reduction in predicted FEV1% and FEV1/FVC in patients with at least two food allergies [52]. Yet, Zicari AM et al. showed a reduction in maximal expiratory flow (MEF) 50 and FEV1 values in a group of children with allergies to both inhalants and foods compared to those only allergic to inhalants [53].

#### 2.3.2. Food Allergy and Asthma Exacerbations

In asthmatic children, the presence of food allergies is a significant risk factor for life-threatening asthma exacerbations and admissions to pediatric intensive care units. Asthmatic patients with food allergies have a higher risk of hospitalization, asthma-related morbidity, and mortality than patients without food allergies. In some children, cofactors such as exercise are necessary to induce asthma exacerbations and anaphylaxis [54]. Simpson AB et al. found an increase in hospitalizations for asthma symptoms in patients with a peanut allergy, as well as an increase in steroid use [55]. A higher morbidity rate than the asthma-only population was also found in other studies performed with a larger population and a wider age range [56]. In particular, in allergic subjects with asthma, severe exacerbations seem to be much more frequent than in the asthmatic-only population: Vogel NM et al. found that patients with emergency room visits for asthma exacerbation had a 7.4 times higher probability of having an allergy to at least one food item, compared to patients who underwent ambulatory evaluations for asthma symptoms [57]. Accordingly, Roberts G et al. found that 50% of patients who accessed the emergency room for asthma exacerbations had food allergies, compared to only 10% of patients who only had asthma [58]. In food-allergic patients with acute asthma exacerbation, it should be considered that symptoms can be triggered by the offending food and, therefore, the administration of intramuscular adrenaline can be necessary.

#### 2.3.3. Anaphylaxis and Severe Reactions to Food in Asthmatic Children

Several studies have assessed whether asthma is associated with an increased severity of symptoms triggered by food allergens. Controversial data on the increased risk of severe reactions during oral food challenges in patients with asthma have been reported [59,60]. The comorbidities of patients with fatal anaphylaxis have been studied and the presence of asthma was highlighted in most of the deceased. Bock et al. reported that only 1 patient of the 64 who died from anaphylaxis induced by the ingestion of offending food did not have asthma [61]. In the study by Calvani et al., asthma increased the risk of wheezing and respiratory arrest during allergic reactions [62]. In a survey of 1094 patients allergic to peanuts or tree nuts, life-threatening bronchospasm was more likely in patients with severe asthma and less likely in patients with milder asthma [63]. On the contrary, a large analysis conducted on fatal anaphylaxis in the U.S. described the presence of asthma in only 16% of patients who died from food anaphylaxis [64]. Anagnostou et al. could not identify asthma as a risk factor [65]. Finally, a recent meta-analysis [66] found no evidence that asthma was a risk factor for the severity of allergic reactions to foods. This association was found only in retrospective studies.

## 3. Sensitization and Exposure

Primary food sensitization can occur through the intestinal route because in infants, both the intestinal barrier [67] and the immune system are immature. Moreover, it can occur through the skin. In children with atopic dermatitis, where the skin barrier is more permeable, the topical application of ointments with peanut oil has been related to a significant increase in the frequency of peanut allergy. In mice, early feeding with food allergens is associated with food tolerance; instead, the exposure of inflamed skin to food allergens causes sensitization that may be related to an allergic gastrointestinal reaction when the food is eaten (“the double exposure” hypothesis). Many studies underline that in high-risk children, a peanut allergy may be prevented by introducing peanuts at ages 4 to 11 months [12]. The ingestion of the culprit food is also known to induce respiratory symptoms such as nasal obstruction, cough, rhinitis, bronchospasm, or laryngeal edema. This is in line with the observation that in mice, epicutaneous sensitization to ovalbumin induces both bronchial eosinophilia following ovalbumin inhalation and hyperresponsiveness to methacholine [68]. Sensitization to foods can also develop through the respiratory route. Many reports have shown that children who have never ingested peanuts and shrimp can experience clinical hypersensitivity reactions including asthma if they inhale these food particles [12]. An asthma attack induced by aerosolized food allergens is usually associated with the steaming of cooked milk or seafood [69]. There are also cases such as baker’s asthma, where continuous environmental contamination with flour can induce frequent asthmatic symptoms due to inhaled wheat proteins; therefore, an accurate history is always necessary [70]. A diagnostic work-up for food allergies, including IgE tests and the avoidance of exposure, should be performed when there is a history of asthmatic episodes to inhaled foods.

## 4. Molecular Mechanisms of Allergic Reactions

### 4.1. Mechanism of Allergic Airway Reactions

Asthma can be categorized into Th2-high and Th2-low types, but mixed airway inflammation is also possible (Table 1) [71,72]. In Th2-high asthma, two major phases are involved: sensitization and challenge. During sensitization, allergens are processed by antigen-presenting cells and presented to Th2 cells, which secrete cytokines like interleukin (IL)-4, IL-5, and IL-13. IL-4 and IL-13 activate B cells, leading to i-E (IgE) production, which binds to mast cells. In the challenge phase, when the same allergen re-enters the airway, it binds to IgE on mast cells, triggering the release of mediators such as leukotrienes, histamines, and interleukins. These cause airway smooth muscle irritation, leading to bronchoconstriction [73]. IL-5 also promotes eosinophil production, and these eosinophils release further mediators that worsen inflammation and contribute to asthma symptoms, such as bronchospasms [74]. IL-13 enhances smooth muscle contraction and stimulates the production of mucins by epithelial cells, contributing to mucus overproduction and airway fibrosis [75]. Epithelial cells also release cytokines like TSLP, IL-25, and IL-33 in response to injury or infection. These cytokines activate innate lymphoid cells (ILC2), which then release Th2 cytokines that worsen lung inflammation [73]. IL-33 may also play a role in mast cell activation and airway muscle migration, affecting the asthma phenotype [76]. T-Helper (Th9) cells, which secrete IL-9, are also involved in Th2 inflammation by promoting mast cell accumulation and activating other inflammatory cells like macrophages. IL-9 can further amplify type 2 inflammation through chemokine secretion that recruits eosinophils and T cells into the lungs [77]. In addition to Th2 inflammation, IL-17 plays a key role in Th2-low asthma. IL-17 cytokines are produced by various cell types, including Th17 cells, CD8+ T cells, and ILC3 cells, and have been found in higher concentrations in severe asthma. IL-17 can induce the release of neutrophil chemoattractants, which drive neutrophil infiltration into the lungs. IL-17A, in particular, enhances smooth muscle contraction and airway remodeling, contributing to asthma’s hyperreactive nature. However, IL-17 cytokines may also have protective effects on airway integrity [78,79]. Th1 cytokines, traditionally associated with phagocytic activity, may also contribute to asthma. TNF-α, a Th1 cytokine, has been implicated in severe asthma and neutrophilic inflammation. It synergizes with IL-17 to recruit neutrophils and potentially promotes the production of Th2 cytokines. TNF-α also enhances smooth muscle contraction, contributing to airway hyperresponsiveness (AHR) [79,80]. Additionally, Th1 cytokines like IFN-γ and IL-1 can upregulate CD38 expression, which increases intracellular calcium signaling in smooth muscle cells, leading to AHR [81,82].

### 4.2. Mechanism of Allergic Food Reactions

Immunological tolerance usually results from the assumption of foods by ingestion and is more evident in the first months of life. The amount and variety of foods reaching the bowel, the integrity of intestinal permeability, and the immune response of gut immune cells influence the modulation of this process [83]. Defects in the intestinal setting such as alterations in gastric pH and intestinal microbiome, and decreased intestinal surface increase the risk of food allergies [84,85,86]. Moreover, the penetration of antigens through skin exposure is also involved in food sensitization, especially in children with atopic dermatitis. [87]. The integrity of intercellular barrier proteins of tight and adherence junctions can be damaged by environmental irritant biological and chemical molecules like protease enzymes of allergens, detergents, tobacco, ozone, particulate matter, diesel exhaust, nanoparticles, and microplastics [88]. Skipping the cleavage mechanism and activating the inception of Th2 immunity, the intact food allergens lead to the secretion of epithelial-derived cytokines, IL-25, TSLP, and IL-33. These mediators (Table 1) stimulate the production of IL-13 and IL-5 through the innate immune system, with the induction of Th9 cells producing IL-9 as a mast cell growth factor and activating the Ig-E response. IL-33, IL-25, and TSLP activate lymphoid type 2 interstitial cells (ILC2) which join to the stimulus in the secretion of IL-13 and IL-4, shifting the differentiation of allergen-specific regulatory T cells into Th2-like cells, with a subsequent breakdown of immunological tolerance for the specific food antigen and the induction of the allergic response, activating the IgE synthesis [89,90,91]. In IgE-mediated allergic reactions, IL-4, IL-5, and IL-13 produced by mast cells, basophils, dendritic cells, Th2 lymphocytes, and ILC2 directly stimulate B cells in the production of IgE, eosinophils in the secretion of IL-6, IL-8, IL-10, and IL-13, leukotrienes through granulocyte–macrophage colony-stimulating factors and eotaxins, and macrophages during differentiation in the M2 subtype [92,93]. Despite the still unknown mechanisms leading the pathogenesis of non-IgE-mediated food allergies, shared pathogenetic and epidemiological elements are detectable. For instance, in atopic dermatitis alterations in fibroblasts which influence the increased production of CCL2 and CCL19 chemokines, inflammation involves a major secretion of IL-22 and IL-26 by tissue-resident memory T cells, with a consequent secretion of inflammatory epithelial-derived cytokines such as IL-33, TSLP, IL-17, and IL-19 [94,95,96]. Th2 cells are stimulated also by the proliferation of TSLP. The resulting increased production of IL-4, IL-5, and IL-13 influence the pathogenesis of eosinophilic gastrointestinal pathologies with the aberrant activation of the eosinophilic pathway and a weakening of the epithelium related to a reduction of filaggrin and desmoglein-1 production through epigenetic modifications. The increased transepithelial passage of antigenic proteins is involved in the development of sensitization [97].

### 4.3. The Molecular Link Between Asthma and Food Allergies

Th2 cells are therefore the main drivers of allergic responses and the molecular link between asthma and food allergies. Th2 cells promote the activation, differentiation, proliferation, and class-switch recombination of B cells that produce allergen-specific IgE antibodies. For both asthma and food allergies, a compromised skin barrier is also an important factor: the transepidermal penetration of antigens leads to sensitization. Inflammation resulting from a compromised skin barrier leads to the increased production of alarmins (IL-25, IL-33, and TSLP), produced by epithelial cells (and subepithelial dendritic cells), which are the main contributors to Th2 cell development and stimulate the production of IL-4, IL-5, and IL-13 by innate lymphoid cells and basophils [98]. Epithelial-derived cytokines strongly stimulate Th2 to the disadvantage of Th1 immunity, resulting in a powerful induction of the allergic response. In this way, dendritic cells are activated and can process the allergen by draining it into the lymph nodes. Through interaction with naive T and B cells, dendritic cells stimulate a specific Th2 allergen response, with subsequent systemic effects at distant tissue sites. Th2 cells, together with ILC2, drive type 2 inflammation by producing type 2 cytokines, including IL-4, IL-5, and IL-13. All three of these cytokines are involved in the trafficking of eosinophils to tissues, which are in turn activated by IL-5. IL-4 and IL-13 are the key and central cytokines for type 2 airway diseases, including asthma. They play a key role in B-cell class switching and IgE production, leading to basophil and mast cell degranulation and the subsequent release of pro-inflammatory mediators, as well as barrier disruption and tissue remodeling. IL-4 positively regulates the differentiation of naive Th0 cells into Th2 cells, which in turn produce IL-4, IL-13, and IL-5, thus creating a cyclic effect. IL-4, IL-5, and IL-13 also induce chemokines (e.g., eotaxin-3, thymus-, and activation-regulated chemokine) and vascular cell adhesion molecule 1, which promote the migration and trafficking of inflammatory cells, including eosinophils, to the site of inflammation [99]. IL-13 is involved in goblet cell hyperplasia and mucus production. Regarding food allergies, Turcanu et al., in a study in 2003, showed an abundance of IL-4, IL-5, and IL-13-producing T cells in peanut-allergic donors compared to donors who had overcome their peanut allergy [100] Berin et al., in 2018, but also in subsequent studies, showed a robust Th2 skew of T cells from peanut or egg allergic individuals [101,102]. Chiang et al., in 2018, showed a very reduced frequency of antigen-specific T cells and a lack of type 2 skew in healthy controls without food allergy [103]. Noah et al., in 2019, demonstrated that goblet cells and other secretory cells are involved in the absorption of dietary antigens; these are called goblet cell-associated passages or GAPs. IL-13 regulates these passages, being involved in goblet cell hyperplasia and mucus production. In addition to this transcellular pathway, there is evidence that type 2 cytokines regulate enterocyte tight junctions, allowing the paracellular passage of macromolecules [104]. IL-9 gene expression is also elevated in the gut of individuals with food allergies; it is a cytokine that promotes mast cell expansion and has been shown to be derived from Th2 cells in food allergies [103]. At the pulmonary level, it amplifies the type 2 inflammatory response through chemokine secretion that recruits eosinophils and T cells into the lungs.

## 5. Treatments and Therapies for Comorbid Asthma and Food Allergies

The goal of standard asthma management is to achieve good symptom control and reduce the risk of exacerbations, as well as to effectively treat asthma attacks. The long-term treatment of asthma depends on several factors, including age, symptoms, asthma triggers, lung function tests, the frequency of symptoms, and a history of severe exacerbations requiring systemic glucocorticoids. Long-term controller medications, including cromolym, inhaled corticosteroids (ICSs), and leukotriene antagonists aim to reduce the airway inflammation that leads to symptoms [105]. ICSs are the cornerstone of asthma therapy. Their molecular mechanism favors the suppression of inflammation through various pathways, including the activation of anti-inflammatory genes and the switching-off of inflammatory genes. In particular, the glucocorticoid receptor (GR) is translocated to the nucleus via the nuclear import protein importin-alpha. The transcription factor GATA3 in Th2 lymphocytes regulates the expression of Th2 cytokines (IL-4, IL-5, and IL-13), which orchestrate allergic inflammation. This pathway is involved not only in the pathogenesis of asthma but also in food allergies. GATA3 is also imported into the nucleus via importin-alpha, but the activated GR takes precedence, preventing the nuclear import of GATA3 and thus rapidly inhibiting the expression of Th2 cytokines and suppressing allergic inflammation [106]. For controller therapy, when ICSs alone are not sufficient, the combination of long-acting beta-2 agonists (LABAs) and inhaled corticosteroids (ICSs) is the most commonly used. LABAs act on beta-2 receptors and have a bronchodilatory action, but they can also complement the action of ICSs by activating the GR themselves, enhancing the transcription of anti-inflammatory mediators [107]. Cromolyn, ICSs, and long-acting bronchodilators, but not leukotriene antagonists, may theoretically interfere with reactions to food since they reduce bronchial hyperactivity. The degree of this effect is unclear. The treatment of acute asthma attacks is primarily based on the use of inhaled short-acting beta-2 agonists (SABAs). When the response to treatment is unsatisfactory, ipratropium bromide, an anticholinergic agent that provides bronchodilation through smooth muscle relaxation, is also used in acute attack [108], and systemic glucocorticoids, with their anti-inflammatory action which effectively reduces airway edema and secretions associated with acute asthma exacerbations [109], are used. In severe attacks, intravenous magnesium sulfate [110] and intravenous SABAs can be given. Drugs for treating asthma attacks may potentially inhibit the onset of asthma during an allergic reaction to food. The value of this prevention is unclear. International asthma guidelines advise against the administration of epinephrine (adrenaline) in acute asthma attacks unless associated with anaphylaxis or angioedema. Epinephrine is a non-selective alpha- and beta-adrenergic agonist that causes bronchodilation through the stimulation of β2-adrenergic receptors, resulting in the relaxation of the bronchial smooth muscle; the alpha-agonist effects of epinephrine may also reduce airway edema [111]. A meta-analysis of RCTs, including epinephrine versus a selective β2-agonist for acute asthma, by Baggott et al. found that epinephrine and selective β2-agonists have similar efficacy in acute asthma attacks, but epinephrine has a worse side effect profile. There is no evidence of benefit from the use of epinephrine in addition to selective β2-agonists in acute asthma, but the available evidence is insufficient to exclude a clinically important benefit. Further studies will be needed to determine whether the addition of intramuscular epinephrine to SABAs in the treatment of asthma attacks significantly modifies the risk of death and treatment failure [112].

The treatment of food allergies is currently based on the avoidance of culprit foods and the prescription of self-injectable adrenaline for patients with a history of severe reactions. This has a significant impact on the quality of life of the patient and the family, linked to the need to constantly monitor the patient’s diet and the anxiety of being exposed to severe reactions due to accidental ingestion of the allergenic food [113,114].

## 6. Novel Treatments

Recently, the increase in allergic diseases has led the scientific community to develop further treatments that may have the potential to influence the natural history of the allergic disease by modulating the immune mechanisms responsible for the hypersensitivity reaction and the evolution of the allergic disease.

### 6.1. Oral Immunotherapy to Foods in Children with Asthma and Food Allergies

For some children with IgE-mediated food allergies, oral immunotherapy (OIT) can be an alternative to avoidance of the culprit food. OIT to foods is a multi-step approach in which one or more food allergens are given in progressively increasing doses over a specified time interval [115,116]. OIT protocols are not yet standardized, and involve an initial dose escalation phase, home OIT dosing, dose escalation (every two weeks), and a maintenance phase with home-dosing every day [117]. Unfortunately, OIT cannot be considered a cure for food allergies, but it can reduce the risks associated with accidental exposures to the offending foods, especially cow’s milk, eggs, and peanuts, with the perception of a benefit on the quality of life [118]. Adverse reactions have been commonly reported during OIT. The most common adverse reactions are mild, consisting of gastrointestinal symptoms, oral allergy syndrome, and cutaneous reactions including rash, urticaria, and/or angioedema [119]. Life-threatening reactions needing intensive care are exceptional. Suboptimal asthma control is a risk factor for adverse events [120]. Therefore, when planning OIT, regardless of severity, asthma is one of the factors that should be carefully considered [121,122]. Severe and/or uncontrolled asthma is a contraindication to OIT according to available guidelines [121]. Patients should achieve good asthma control before starting OIT. Asthma may increase the probability of allergic reactions, both in frequency and severity, during OIT. Several studies have highlighted relative risk factors associated with an increase in adverse events. In a study of 130 children aged 5 to 18 years receiving OIT (80 cow’s milk OIT and 50 egg OIT) [123], three cases of life-threatening anaphylaxis occurred. They shared common characteristics: puberty, persistent asthma with suboptimal control, elevated specific IgE levels, and poor adherence to oral therapy and/or asthma treatment. In addition, of these three subjects, two patients undergoing cow’s milk OIT required invasive ventilation with intubation during hospitalization, while the third undergoing OIT for eggs required noninvasive ventilation. A large retrospective analysis showed that a higher rate of adverse events during peanut OIT occurred in children with asthma and allergic rhinitis; in another retrospective study, intermittent asthma was associated with an increased rate of severe reactions to peanut OIT that were treated with epinephrine during dose escalation [124,125]. In a prospective longitudinal trial, subjects aged 5 to 18 years old received OIT to cow’s milk without premedication [126]. In this study, the most frequent reactions in patients were asthma, hoarseness, diarrhea, mild hypotension, or dysrhythmia (classified as grade 4 reactions). Isolated mild asthma was the most frequently observed symptom (76% of grade 4 reactions). Fifty-four children received salbutamol and nine received epinephrin. Thirty-two percent of reactions developed with dose escalations during the induction phase and 12% of reactions occurred in association with cofactors, involving 47 children (58%), of whom 6 had asthma exacerbation. Staden U et al. [127] showed that asthma was related to the persistent reaction group, but it did not influence the continuation of OIT to cow’s milk. Factors linked with the severity and persistence of cow’s milk allergy (higher cow’s milk specific IgE and SPT, co-existing asthma/rhinitis, and low eliciting cow’s milk dose) [128,129,130] are also those associated with failure and a higher rate of reactions in OIT to cow’s milk [131]. Therefore, it appears that cow’s milk OIT may contribute to decreased asthma control. In a retrospective report of patients aged > 3.7 years who underwent OIT for food allergies [132] cow’s milk OIT was significantly associated with epinephrine-treated reactions. In these patients, there was also a slightly higher prevalence of asthma (53.1% versus 46.2%), which might explain, in agreement with other studies, the greater severity of the reactions. Furthermore, in asthmatic children allergic to cow’s milk, significantly worse treatment results were found, with only 18.2% reaching complete desensitization, in contrast with 73.9% during OIT for other foods. Therefore, before starting OIT it is necessary not only to evaluate the severity and control of asthma but also to discuss the risk of reaction to OIT [133,134]. As an alternative to oral administration, other routes of allergen administration are being studied, in particular, the sublingual [135] and epicutaneous routes. So far, studies conducted with milk, hazelnuts, peanuts, and peaches have shown that sublingual immunotherapy (SLIT) is safer than OIT, as 99% of the reported reactions are mild within the oral cavity. Moreover, during SLIT, systemic reactions such as asthma are uncommon, with one study observing a 0.2% rate of reactions. However, SLIT is less effective than OIT. Several trials have shown that modification in the threshold doses of reactivity to the offending food is lower with SLIT than with OIT [136,137,138]. Studies conducted on epicutaneous immunotherapy (EPIT) for cow’s milk and peanuts demonstrated that it has a lower frequency of adverse reactions, with higher compliance compared to OIT. The adverse reactions are usually mild and localized at the site of patch application. During EPIT, systemic adverse effects such as asthma are rare since a low amount of the allergen administered through the epicutaneous route is absorbed [139,140,141]. The REALISE (Real Life Use and Safety of EPIT) randomized controlled trial [142], which included a good proportion of highly atopic participants, found that most of the reported adverse events were mild (82.7%) or moderate (36.9%). However, the effectiveness of EPIT is less than OIT.

### 6.2. Biologics in Children with Asthma and Food Allergies

Considering the current limitations of both allergen avoidance and OIT, novel therapies for food allergies are being studied. Biologics is an adjuvant therapy that has good efficacy [143,144]. Over the past two decades, they have been shown to be highly effective in targeting immunological mediators of Th2 inflammation in allergic asthma. In the last decade, several studies have assessed the use of biologics for severe asthma and for reducing allergic inflammatory pathways in food allergies.

#### 6.2.1. Omalizumab

Omalizumab (OMA) is an anti-IgE recombinant humanized monoclonal antibody (mAb). OMA both as monotherapy and, as an add-on to OIT (OMA plus OIT), has been investigated by several research groups [145,146]. OMA binds to the CHε3 region of IgE. Thus, IgE cannot bind to the FcεRI and FcεRII receptors on mast cells and basophils that release the mediators responsible for the allergic response [147]. Subcutaneous OMA was the first biologic authorized for severe asthma in children aged ≥ 12 years by the U.S. Food and Drug Administration (FDA) in 2003, and by the European Medicines Agency (EMA) in 2005. Later, in 2016, it was approved for children aged over 6 years. OMA has already been authorized for nasal polyposis and chronic urticaria. OMA significantly decreased the number of exacerbations in asthmatic children with demonstrated safety [148,149,150,151,152,153,154,155]. The useful effects were sustained up to 6 years post-treatment [156]. Furthermore, several studies have shown that subcutaneous immunotherapy for seasonal allergies, in combination with OMA, reduces symptoms much more than subcutaneous immunotherapy alone [157,158]. Bozek et al., in their study, showed that in patients with asthma due to house dust mites, the combination of subcutaneous immunotherapy plus OMA reduced not only symptoms but also the daily dose of inhaled corticosteroids needed to control asthma exacerbations, more than OMA alone or subcutaneous immunotherapy alone [159]. Due to its mechanism, a potential role of OMA in the therapy of IgE-mediated food allergies was later hypothesized [160,161,162]. Rafi et al. [163] treated patients who were allergic to fish, shellfish, peanuts, tree nuts, eggs, soybeans, and wheat with OMA 22 asthmatics. They found that allergic reactions following the accidental ingestion of offending foods were absent or reduced compared to previous reactions induced by a similar quantity of the food in question. Nilsson et al. described five children with an IgE-mediated severe milk allergy who passed an oral milk challenge after 16–32 weeks of OMA treatment. The basophil allergen threshold sensitivity (CD-sens) was helpful in predicting the food challenge outcome [164]. Dahdah et al. administered OMA to a boy with severe asthma and anaphylaxis to different foods following selective IgE apheresis. After 40 days of treatment, he partially or fully tolerated baked milk, hazelnuts, soy, and chicken, with a better quality of life [165]. In an observational, real-life study of 15 children with severe, uncontrolled asthma receiving OMA and with multiple food allergies, Fiocchi et al. [166] assessed the food allergen thresholds and the type of immediate reactions related to food ingestion after 4-month OMA treatment. It was found that food allergen thresholds increased up to 8.6 times compared to the baseline challenge. After the course of OMA, the food challenge result was negative in 70.4% of subjects. All offending foods that were tested were successfully introduced into the diet of nine patients. Two children were able to tolerate at least one offending food, and four partially tolerated the offending food items. Their asthma control improved. There was a significant increase in quality of life, as expressed by the Pediatric Quality of Life Inventory (PedsQL) [166]. The treatment dosage of OMA was based on body weight and IgE levels, according to a modified dosing algorithm for asthma, as already used in other studies [167,168,169]. Thus, patients with high baseline IgE levels, potentially excellent candidates for therapy with OMA, would be excluded from the study. In a phase 3 multicenter trial, Wood et al. [170] included patients aged 1–55 years, with a median total IgE level of 700 IU per milliliter, and multiple food allergies with positive food challenges to peanuts and at least two foods among cashew, egg, cow’s milk, walnut, wheat, or hazelnut. Patients were randomized to receive OMA or a placebo every 2 to 4 weeks for 16 to 20 weeks, followed by a second food challenge. The primary outcome was tolerance of ≥600 mg of peanut protein. OMA for 16 weeks greatly enhanced the reaction threshold to the ingestion not only of peanuts (67% vs. 7% *p* < 0.001) but also of cashews, egg, and cow’s milk than the placebo. The open-label extension phase showed that a 24-week treatment maintained a stable or increased challenge threshold for most patients. The adverse event rate was similar in the two groups and the quality of life of participants and caregivers did not change [170]. The aforementioned studies show that OMA increases the amount of the offending food that is needed to trigger severe systemic reactions, including asthma and anaphylaxis after inadvertent exposure. Based on such data, the FDA has approved OMA monotherapy in patients aged 1 year and older with IgE-mediated food allergies for decreasing severe allergic reactions, including anaphylaxis, following the accidental intake of one or more offending foods. The FDA also recommends maintaining an avoidance diet during therapy with OMA since OMA should not be considered a cure for food allergies. Moreover, it is not certain whether OMA may reduce the severity of a reaction that occurs after ingesting a quantity of food greater than the tolerated one. Further studies will also be needed to better understand the reasons for treatment failures, as well as the possibility of modifying the natural history of the disease with early and prolonged OMA use. Finally, several studies have highlighted that the combination of OMA plus OIT treatment may decrease the immediate side effects of OIT and enhance the immunomodulatory effect of OIT [167,168,169,171,172,173,174]. When OMA was added to OIT for peanut and milk allergies, both more rapid desensitization and improved safety with fewer adverse reactions at the same doses were seen [169,171,174]. Andorf et al. [175] studied 36 weeks of OIT treatment with OMA (n = 36) or a placebo (n = 12) in children with allergies to 2–5 foods. A double-blind, placebo-controlled food challenge to ≥2 g of food protein for allergies to two or more foods was passed by 83% of children in the OMA group and 33% in the placebo group. Adverse events were less common in the OMA group than in the placebo group (27% vs. 68%; *p* < 0.0082) and no serious or severe (≥grade 3) adverse event occurred [175]. Even if sustainability of desensitization was not observed for milk OIT (48% OMA group vs. 37% placebo group) [167] and in multi-OIT (55% vs. 45%) [168], it was seen in peanut OIT (74% vs. 12%) [169]. Moreover, the lowest effective dosage of OMA, in terms of a single dose and duration of pre-OIT and OIT treatment, is still unclear. The optimal dosing of OMA in association with OIT protocols needs further investigation considering the high cost of the drug [173] and data showing that the OMA dosage should be adjusted only according to the body weight of the patient, without considering IgE levels [176].

#### 6.2.2. Other Biologics

Dupilumab IgG4 human mAb binds the IL-4 receptor alpha subunit (IL-4Rα) and inhibits IL-4 binding to the receptor [177]. It suppresses the development of the IL-13 Ra 1/IL-4Ra heterodimer receptor complex for IL-4 and IL-13, which are involved in T2 activation in several allergic diseases, comprising food allergies. Dupilumab is currently approved for moderate to severe atopic dermatitis in patients 6 months or older by the FDA and the EMA [178,179], and for severe eosinophilic asthma, chronic rhinosinusitis with nasal polyps in adults, and eosinophilic esophagitis in patients 12 years or older. In asthmatic patients, dupilumab is effective in decreasing the dose of oral glucocorticosteroids and the number of exacerbations, and in improving pulmonary function [180]. Dupilumab is now being studied for food allergies [181]. The first study describing the efficacy of dupilumab in food allergies was published by Rial et al. in 2019, who reported that a woman was desensitized to corn and nuts after 3 months of dupilumab treatment [182]. Currently, ongoing studies assess adverse events and the effectiveness of dupilumab as an adjunct to peanut OIT, and as a monotherapy for peanut allergies [183,184], and in a trial that compares OMA, dupilumab and a placebo were added as treatments during multifood OIT [185,186,187]. Moreover, Abdel-Gadir et al. showed that dupilumab suppresses the function of regulatory T cells during peanut exposure in allergic patients, highlighting the potential modulatory effect of the anti-IL4 antibody on food allergies [188]. IL-5 is another studied cytokine with a crucial pathogenic role in mediating the Th2 response. The molecule is produced by many cells, including eosinophils, Th2 cells, mast cells, natural killer T cells, and ILC2s. IL-5 binds to the IL-5Rα receptor on the eosinophil surface and induces the formation of the IL-5/IL-5Rα/βc ternary complex; this leads to the differentiation, the recruitment, and the degranulation of eosinophils [74,189]. Currently, mepolizumab, reslizumab, and benralizumab are biologics that target the IL-5/IL-5R pro-eosinophilic axis. Mepolizumab and reslizumab are mAbs that work against IL-5, and benralizumab is a mAb that works against the α subunit of the interleukin-5 receptor on eosinophils. These biologics are authorized for eosinophilic asthma, eosinophilic granulomatosis with polyangiitis, and eosinophilic esophagitis [190,191,192]. Their successful use in eosinophilic gastrointestinal disorders is promising for a potential application in the treatment of food allergies. The pathogenesis of eosinophilic gastrointestinal disorders, mainly eosinophilic esophagitis, has genetic and environmental factors similar to those of IgE-mediated food allergies [193]. Tezepelumab is an anti-TSLP mAb that inhibits the link to the TSLP receptor. It is approved for severe asthma. It might be potentially helpful in food allergies [194], since TSLP induces the expression and differentiation of Th2, the production of IL-4, and the activation of ILC2. Ligelizumab is a humanized, recombinant, anti-IgE monoclonal IgG1K antibody. Its affinity for IgE is higher than OMA. Thus, it is more effective in blocking the IgE-dependent release of mast cell mediators [195]. A phase 3, 52-week trial on ligelizumab monotherapy for patients with peanut allergy is ongoing.

## 7. Conclusions

Several lines of evidence indicate that children with allergic diseases are susceptible to developing other allergic conditions over time that share a common T2 inflammatory pathophysiological mechanism. Among allergic multimorbidities, food allergies often precede the occurrence of asthma. A greater understanding of the frequent co-existence of asthma and food allergies represents a key factor in improving their management. Several aspects are important. Asthma raises the risk of severe allergic reactions to offending foods. On the other hand, morbidity or mortality due to asthma is increased in asthmatic patients with food allergies. Given the higher risk of severe asthma attacks and anaphylaxis, patients with asthma and food allergies should receive action plans providing information and instructions on how manage acute episodes. The control of asthma influences access to and the course of OIT to foods, which is offered for increasing tolerance to specific foods. Of note, the use of OMA, already known for its benefits in asthma control, has been recently extended to patients with food allergies. OMA is effective as a monotherapy or in association with OIT for increasing the dose of foods that induce symptoms in order to avoid severe reactions due to inadvertent ingestion. Further studies are warranted to clarify whether algorithms can be detected to stratify the risk of clinical evolution in allergic patients and whether biologics can modify the natural history of allergic diseases. The costs of biologics, allergen immunotherapy, and OIT considerably go beyond the annual direct cost of medication and healthcare for asthma or food allergies. Access to these treatments is hampered by costs for patients and health services, not only for medication but also for follow-up visits that increase the absenteeism and presenteeism of patients and caregivers. Thus, the use of biologics is reserved for the most severe cases. This limitation in real life also applies to OIT and allergen immunotherapy against inhalants in asthmatic patients, even if it is recommended in less severe cases. Some realistic and safe developments may reduce costs and enhance adherence of patients. They include the self-administration of biologics, mobile health or telehealth programs for monitoring patients, and the transition of treatment management to the primary care setting. Of note, there are areas where further research is needed. Trials are warranted to determine the long-term safety of biologics, and whether biologics can modify the natural history of allergic diseases. Studies are needed to clarify whether algorithms can be created to stratify the risk of clinical evolution in treated patients. In addition, more studies should highlight the efficacy of allergen immunotherapy combined with biologics and that of biologics compared to OIT or sublingual immunotherapy for food allergies. Finally, it would be of interest to know whether biologics may improve disease control in mild–moderate asthma and in non-anaphylactic reactions to foods.

## Figures and Tables

**Table 1:** Cells and mediators involved in molecular pathways of asthma and food allergies [71,72]. B, B lymphocytes; CCL2/19, chemokine ligand 2/19; CRTH, chemoattractant receptor homologous expressed on Th2 cells; IgE, immunoglobulin E; ILC2/3 lymphoid type 2/3 interstitial cells; IL, interleukin; IFN-γ, interferon-gamma; NK-T, natural killer T cell; SIGLEC-8, sialic acid-binding Ig-like lectin 8; Tc2/17, T cytotoxic lymphocyte type 2/17; TGF-beta, tumor growth factor; Th-2/17, T-helper lymphocytes 2/17; TSLP, thymic stromal lymphopoietin; TNF-α, tumor necrosis factor-alpha.

	**CELLS**		**MEDIATORS**	
ASTHMA	Th-2 B cells IgE ILC2 ILC3 Tc2 Th-17	Tc17 NK-T Mast cells Neutrophils Basophils Eosinophils	IL-4 IL-5 IL-9 IL-13 IL-17 IL-22	IL-23 IL-31 TNF-α IFN-γ TSLP Perforines
FOOD ALLERGY	Th-2 B cells IgE ILC2 Tc2	NK-T Mast cells Neutrophils Basophils Eosinophils	IL-4 IL-5 IL-6 IL-8 IL-9 IL-13 IL-17 IL-19 IL-22 IL-26 IL-31	IL-31RA IL-33 TSLP CCL2 CCL19 Eotaxi-3 CRTH2 SIGLEC-8 TGF-beta TNF-α
